# Exploring the Relationship Between Serum Phosphate Levels and Carotid Intima-Media Thickness in Chronic Kidney Disease Patients: A Correlational Analysis

**DOI:** 10.7759/cureus.60308

**Published:** 2024-05-14

**Authors:** Prabhakar K, Anitha A, Sanjana M, Roshan Prasad

**Affiliations:** 1 Medicine, Sri Devaraj Urs Medical College, Kolar, IND; 2 Medicine and Surgery, Jawaharlal Nehru Medical College, Datta Meghe Institute of Higher Education and Research, Wardha, IND

**Keywords:** b-mode ultrasonography, serum phosphate, cimt, atherosclerosis, chronic kidney diseases

## Abstract

Background

Compelling observational data suggest that heightened levels of fasting blood phosphate are linked to a higher likelihood of cardiovascular disease, spanning across both the general populace and individuals grappling with chronic kidney disease (CKD). This study aimed to explore the possible correlation between carotid intima-media thickness (CIMT) and blood phosphate levels among those afflicted with chronic renal dysfunction.

Objective

The primary goal of this study is to determine the potential association between blood phosphate levels and CIMT in patients with CKD.

Methodology

In the department of nephrology, prospective research was conducted among patients who had a history of CKD. A total of 30 patients were included, with 20 males and 10 females. Every case had a thorough physical examination and history. Every patient underwent a laboratory evaluation, which included measurements of the CIMT and renal function testing. At a distance of 1 cm from the carotid bulb, the CIMT was measured using B-mode ultrasonography. After compilation, the data were examined.

Results

The majority of the patients, according to this study, were male and over 50 years old. The Stage II patients in the study had a higher mean systolic blood pressure; however, the difference was not statistically significant. Patients with Stage V (D) disease exhibited higher diastolic blood pressure, but not statistically significant. An increase in the mean serum creatinine level that was statistically significant was linked to Stage V (D) renal disease. A higher mean blood urea was linked to Stage V (D) sickness; however, this relationship was not statistically significant. There was no statistical difference in the mean serum calcium levels between the different stages of renal disease. Higher mean blood phosphate levels were linked to Stage III renal disease, but not in a statistically meaningful way. Although it was higher in Stage IV kidney disease, the mean CIMT was not statistically significant between the stages of renal illness.

Conclusions

Although a positive correlation was shown, a direct relationship between serum phosphate levels was not established by this investigation. The severity of renal disease has been demonstrated to correlate with elevated serum phosphate levels.

## Introduction

Chronic kidney disease (CKD) strongly predicts cardiovascular disease [1]. The last stage of chronic renal failure (CRF), an irreversible loss of renal function, is end-stage renal disease (ESRD) [2]. ESRD patients have a 10-20 times higher risk of dying than the general population of the same age and gender. Stroke and cardiovascular disease are this population's leading causes of death [3-5]. The yearly mortality rate from cardiovascular diseases among hemodialysis (HD) patients is approximately 9%, which is 30 times higher than that of the general population [6]. Cardiovascular disease mortality and morbidity are higher in HD patients than in the general population, even among those under 45 [7,8].

Recent findings indicate that the thickness and stiffness of the arterial walls indicate more advanced arteriosclerosis in patients with CRF [9,10]. In the general population, smoking cigarettes, hypertension, and hyperlipidemia are the main risk factors for progressive arteriosclerosis [11]. It is common for serum phosphate levels to remain within the standard laboratory range until a very advanced stage of CKD. As renal function declines, parathyroid hormone (PTH) levels increase, and 1,25-dihydroxy vitamin D levels decrease. There are strong observational data to show that individuals with CKD, as well as the general population, have a higher risk of cardiovascular disease when their fasting blood phosphate level is raised. Serum phosphate was therefore found to have a strong connection with carotid intima-media thickness (CIMT) and even with mortality in individuals with adequate renal function, even when typical cardiovascular risk indicators were disregarded [12,13].

While a higher serum phosphate concentration is a significant risk factor for vascular calcification, further study is needed to identify whether blood phosphate level is a risk factor for growing arterial wall thickness in individuals with CKD [14]. This investigation aimed to assess the relationship between the CIMT, a marker of atherosclerosis, and serum phosphate levels.

## Materials and methods

Study design and participants

A prospective study conducted at the Department of Nephrology at the Sri Devaraj Urs University of Medical Sciences, Kolar's Shri RL Jalappa Hospital, included patients with CKD. The calculation of the sample size was done using the correlation coefficient. Keith et al. [15] showed a 0.911 correlation coefficient with CIMT at an 80% power level and 5% significance level. The estimated sample size of six was expanded to 30 for statistical reasons. Consent was obtained or waived by all participants in this study. The study obtained approval from the Central Ethics Committee of Sri Devaraj Urs Academy of Higher Education and Research on Human Subjects with approval number SDUAHER/KLR/R&D/CEC/S PG project/58/2024-25.

Selection Criteria

The study included patients with a history of CKD who were older than 18 years. The research excluded patients with acute renal problems, those who had undergone carotid surgery in the past, and those who were pregnant.

Data sources and variables

The medical history of the patient was obtained and recorded, encompassing details on diabetes mellitus, smoking history, renal failure causes, and past and familial histories of cardiovascular disease. Anthropometric measurements, including height, weight, and BMI, were conducted.

All significant metrics were recorded, such as respiratory rate, pulse rate, and blood pressure. On fasting blood samples, the following parameters were measured: serum levels of calcium, phosphorus, uric acid, hemoglobin, albumin, creatinine, and lipid profile (triglyceride, total cholesterol, high-density lipoprotein, and low-density lipoprotein). Approximately 1 cm away from the carotid bulb, the CIMT was measured using B-mode ultrasonography. During the ultrasonography procedure, the patients were placed in a supine position while their necks were extended to assess the bifurcation point, common carotid, and proximal internal carotid arteries.

Statistical analysis

The master graphic containing the data was filled out using Microsoft Excel (Microsoft Corporation, Redmond, Washington). The IBM SPSS Statistics for Windows, Version 20 (Released 2011; IBM Corp., Armonk, New York) was used to evaluate the gathered data. Frequency, percentage, and mean analyses were employed to characterize the data in descriptive statistics for categorical and continuous variables. Non-parametric tests, such as the Mann-Whitney U and Kruskal-Wallis tests, were used to determine the significance of two variables if the results did not follow a normal distribution curve. A statistically significant P-value was defined as less than 0.05.

## Results

Table 1 presents the baseline characteristics of the study group. The age distribution indicates that 90.0% of the patients were over 50 years old, while 10.0% were under 50. In terms of gender, 66.7% were male, and 33.3% were female. Among the causes of CKD, glomerulonephritis accounted for 30.0% of the cases, followed by obstructive uropathy (43.3%), hypoplastic kidney (16.7%), and other causes (10.0%). Regarding the stage of kidney disease, 33.3% were classified as Stage III, 36.7% as Stage IV, 13.3% as Stage V without dialysis (ND), and 16.7% as Stage V with dialysis (D).

**Table 1:** Baseline characteristics of the study group ND: without dialysis; D: with dialysis

	Patient Characteristics	Frequency	Percent
Age	Less than 50 years	3	10.0
	More than 50 years	27	90.0
Sex	Male	20	66.7
	Female	10	33.3
Cause of CKD	Glomerulonephritis	9	30.0
	Hypoplastic kidney	5	16.7
	Obstructive uropathy	13	43.3
	Others	3	10.0
Stage of kidney disease	Stage III	10	33.3
	Stage IV	11	36.7
	Stage V (ND)	4	13.3
	Stage V (D)	5	16.7

Table 2 illustrates the biochemical parameters corresponding to the different stages of kidney disease. Although it was not statistically significant, the mean systolic blood pressure was elevated in this study's patients with Stage II illness. Although not statistically significant, patients with Stage V (D) illness had a higher diastolic blood pressure. Stage V (D) renal disease was associated with a statistically significant increase in the mean serum creatinine levels. Although not statistically significant, Stage V (D) illness was associated with a higher mean blood urea. Between the stages of renal disease, there was no statistically significant difference in the mean serum calcium levels. Although not statistically significant, Stage III renal disease was associated with higher mean blood phosphate levels. The mean CIMT was not statistically significant across stages of renal disease; however, it was higher in Stage IV kidney disease.

**Table 2:** Biochemical parameters with stages of the kidney disease P-value<0.05 is considered to be significant ND: without dialysis; D: with dialysis; Sig: significant; NS: not significant

Parameters	Stage III	Stage IV	Stage V (ND)	Stage V (D)	P-value
Systolic blood pressure	133.6 ± 18.3	123.6 ± 11.3	126.0 ± 17.0	126.8 ± 11.7	0.491, NS
Diastolic blood pressure	86.6 ± 8.7	88.4 ± 7.7	88.5 ± 4.4	90.0 ± 7.6	0.874, NS
Serum creatinine	1.82 ± 1.3	1.7 ± 1.0	5.1 ± 3.23	6.6 ± 2.3	0.000, Sig
Blood urea	91.2 ± 36.8	130.3 ± 45.3	79.6 ± 27.5	106.6 ± 46.3	0.118, NS
Serum calcium	1.3 ± 0.3	1.1 ± 0.2	1.0 ± 0.1	1.33 ± 0.2	0.116, NS
Serum phosphate	4.7 ± 1.2	4.3 ± 0.4	4.1 ± 0.6	4.4 ± 0.6	0.595, NS
CIMT	0.1 ± 0.03	0.2 ± 0.3	0.09 ± 0.02	0.14 ± 0.2	0.585, NS

Table 3 displays the correlation between serum phosphate levels and CIMT. The serum phosphate levels showed a Pearson correlation coefficient of .225 with CIMT, with a P-value of .232 (two-tailed), based on a sample size of 30 for both parameters. Conversely, CIMT exhibited a Pearson correlation coefficient of .225 with serum phosphate levels, also with a P-value of .232 (two-tailed) and the same sample size of 30.

**Table 3:** Correlation between serum phosphate levels and carotid intima-media thickness Correlation is considered to be significant at 0.05 level (two-tailed) CIMT: carotid intima-media thickness

Correlations			
		Serum phosphate	CIMT
Serum phosphate	Pearson correlation	1	.225
	Sig. (two-tailed)		.232
	N	30	30
CIMT	Pearson correlation	.225	1
	Sig. (two-tailed)	.232	
	N	30	30

## Discussion

The main goal of this study was to find out how serum phosphate and CIMT relate to each other in patients with chronic renal disease. Cardiovascular disease is one of the main issues for people with chronic renal illness. Compared to people without the illness and, to a lesser extent, individuals with the disease in Stages I and II, adults with CKD in Stages III-V have a noticeably greater burden of cardiovascular risk factors [16]. Patients with Stage III of the illness have a 20-fold higher risk of dying from cardiovascular disease than from ESRD [17,18]. Individuals with CKD are more likely to acquire cardiovascular disease than ESRD.

Hypophosphatemia is often recognized as a predictor of cardiovascular diseases [18]. Phosphate levels are shown to increase with the severity of CKD. In 90% of the cases, the patients were older than 50 years. Approximately 67% of the patients were female. The cause of the patient's CKD was obstructive uropathy. Approximately 36.7% of the individuals had Stage IV renal dysfunction. In contrast to the findings of this study, a study conducted by Arora et al. found that the mean age of patients with renal illness was 45 years [16]. The research conducted by Rahman et al. revealed a higher number of male cases than female cases, with a mean age of 11.45 years, and hypoplastic kidneys are the primary cause of chronic renal disease [19]. The study conducted by Srikanth et al. found that the average age was 60.6 years [20].

The Stage II patients in this study had a higher mean systolic blood pressure; however, this was not statistically significant. Patients with Stage V (D) disease exhibited a higher diastolic blood pressure, but not statistically significant. A statistically significant increase in the mean serum creatinine was linked to Stage V (D) renal disease. A higher mean blood urea was linked to Stage V (D) sickness; however, this relationship was not statistically significant. The mean serum calcium did not differ statistically significantly between the stages of renal disease. Higher mean blood phosphate levels were linked to Stage III renal disease; however, they were not linked in a statistically significant manner. Although it was higher in Stage IV kidney disease, the mean CIMT was not statistically significant between the stages of renal illness.

According to the study conducted by Arora et al., individuals with renal illness had greater levels of serum phosphate, serum calcium, systolic and diastolic blood pressure, and serum phosphate. The blood phosphate levels in patients with renal disease in Stages III, IV, and V differed significantly. However, Arora et al. found that the CIMT varied significantly [16]. Rahman et al. observed, in contrast to the findings of this investigation, that there was a significant difference between the severity of renal disorders and the serum creatinine, blood urea, and serum phosphate levels [19].

The CIMT varied significantly according to the renal disease stage. Serum phosphate levels and CIMT showed a negligibly negative connection [19]. The study conducted by Srikanth et al. found no significant difference in systolic and diastolic blood pressure readings. Serum creatinine and CIMT varied statistically significantly according to the degree of renal disease [20]. No statistically significant link was seen between the CIMT and serum phosphate levels.

Limitations

In addition to providing some valuable outcomes, the study also had a few limitations. Firstly, the sample size was relatively small, with only 30 participants, potentially limiting the statistical power of the analysis. Furthermore, the study was conducted in a single institution, which may restrict the generalizability of its findings. Further studies can be conducted to provide a more comprehensive understanding, considering the individual's lifestyle and other factors that may influence the study parameters.

## Conclusions

Although this study did not definitively demonstrate a direct cause-and-effect relationship between serum phosphate levels and the observed phenomenon, it did uncover a notable positive correlation. The data suggested that as the severity of kidney disease progressed, serum phosphate levels exhibited a corresponding increase. This observation underscores the potential role of serum phosphate as a marker or indicator of kidney disease severity, providing valuable insights into the complex interplay between serum phosphate levels and renal function.
